# Anuran tadpoles inhabiting a fluoride-rich stream: diets and morphological indicators

**DOI:** 10.1016/j.heliyon.2019.e02003

**Published:** 2019-06-26

**Authors:** Favio E. Pollo, Luciana Cibils-Martina, Manuel A. Otero, Mariana Baraquet, Pablo R. Grenat, Nancy E. Salas, Adolfo L. Martino

**Affiliations:** a Ecología, Departamento de Ciencias Naturales, Facultad de Ciencias Exactas, Físico-Químicas y Naturales, UNRC, ruta 36km 601, Río Cuarto, Córdoba, Argentina; b Botánica Sistemática, Departamento de Ciencias Naturales, Facultad de Ciencias Exactas, Físico-Químicas y Naturales, UNRC, Argentina; c Instituto de Ciencias de la Tierra, Biodiversidad y Sustentabilidad Ambiental (ICBIA), UNRC-CONICET, Argentina

**Keywords:** Environmental science, Periphyton, Trophic ecology, Functional traits, Body condition, *Boana cordobae*, Fluoride

## Abstract

We *in situ* assessed the influence of natural fluoride concentrations in lotic freshwater ecosystems on diet and morphology of *Boana cordobae* tadpoles. Two streams were sampled in Argentina: Los Vallecitos stream (LF-LV) and Los Cerros Negros stream (HF-CN) with low and high fluoride levels, respectively. We captured tadpoles of *B. cordobae* in each stream using nets. Body weight (BW), total length (TL) and body condition (BC) of tadpoles was registered. Food items were identified to genus level and assigned to functional traits. Tadpoles showed significant differences in TL between streams, with smaller individuals in HF-CN, while did not show differences in BW and BC. The diet of tadpoles consisted mostly of microalgae. In tadpoles from HF-CN stream the proportion of cyanobacteria was lower than tadpoles from LF-LV. In relation to functional traits, small algae, high profile and colonial algae were more abundant in HF-CN. Algae attached by pads showed a higher proportion in HF-CN diets and stalked algae were more abundant in LF-LV. The differences in TL and diet of tadpoles can be attributed to differences in algal community composition, with genera that are affected by high concentrations of natural fluoride; for example cyanobacteria. The low algal richness registered in HF-CN stream does not affect the physiological state of the tadpoles, possibly because of a higher algal density in HF-CN diets. However, in further studies it would be important to evaluate the population status of *B. cordobae* from the HF-CN, because a small body length of tadpoles could have consequences at the population level.

## 1 Introduction

In recent years, concern about deleterious consequences of fluoride on several organisms has been developed (Zuo et al., 2018) and especially its potential toxicity to aquatic organisms (Shi et al., 2013; Ballarin et al., 2014; Camargo and Alonso, 2017; Zhang et al., 2018). Fluoride is a non-metallic halogen abundant in the environment, distributed only in combination with other elements as fluoride compounds (Camargo, 2003). This mineral may reach freshwater ecosystems from natural sources that include weathering of fluoride minerals (Weinstein and Davison, 2004) and/or anthropogenic sources (Camargo, 2003; Weinstein and Davison, 2004). For these reasons, there is a worldwide concern about the increase of fluoride levels in freshwater ecosystems (Zhang et al., 2018). Alterations occurring in aquatic organism exposed to fluoride have been the topic of several studies, most of them based on laboratory exposures (Camargo, 2003; Shi et al., 2013; Ballarin et al., 2014; Camargo and Alonso, 2017; Zhang et al., 2018). However, there is limited information on the ecotoxicity of fluoride on trophic ecology of anuran tadpoles (Chai et al., 2016a,b). Thus, there is still the need to conduct field test (*in situ*) with real concentrations using native species (Antunes et al., 2008), to generate more realistic and toxicologically relevant data about the effects of fluoride on freshwater ecosystems. This type of study is important because it considers environmental conditions (e.g., ultraviolet light, suspended solids, water velocity and temperature) that cannot be controlled in the laboratory and could be stressors that alter the toxicity effect (Crane et al., 2007).

Some laboratory experiments revealed that fluoride inhibits population growth of some algae, by affecting cell division and photosynthetic capacity (Antia and Klut, 1981; Hekman et al., 1984). Nevertheless, for other algae, fluoride may be a requirement for optimal growth (Oliveira et al., 1978; Joy and Balakrishnan, 1990). Given the importance of microflora in trophic webs as primary producers, adverse effects of fluoride on them may have serious consequences on organisms at higher trophic levels (Franklin et al., 2000; Chai et al., 2016a,b). Decrease (in quantity and quality) of primary producers can alter the herbivory and therefore the stability of the community (Cardinale et al., 2011). Accordingly, several studies have analyzed the diet of tadpoles in environments associated with different degrees of disturbance (Bionda et al., 2012, 2013; Babini et al., 2016). However, few studies have linked the trophic ecology of tadpoles with the lotic environment (Santos et al., 2016) and how this can be affected by the presence of fluoride. Consequently, the aim of this study was to evaluate *in situ* the influence of natural fluoride concentrations in lotic freshwater ecosystems on diet and morphology of *Boana cordobae* tadpoles. We use tadpoles of the endemic species *B. cordobae* distributed only in highlands of central Argentina (Frost, 2018), because a previous study has shown that they consume large amounts of algae (Pollo et al., 2015). Therefore, could be a good bioindicator of the availability of the algal resource offered by the environment (Arana et al., 2003).

## 2 Materials and methods

### 2.1 Study area and site selection criteria

The study was carried out in two streams located in the Sierras Pampeanas of Córdoba, Argentina: Los Cerros Negros stream (32°30′16″S, 64°48′06″W; 1246 m a.s.l.; HF-CN), which flows through granitic rock with high natural fluoride content, and Los Vallecitos stream (32°29′58″S, 64°47′31″W; 1237 m a.s.l.; LF-LV), flowing through metamorphic rock with low fluoride concentration. Geologically, this area is dominated by granitic and metamorphic rock. Granitic rock contains high concentrations of fluorite (CaF_2_), approximately two times as much as host metamorphic rocks and other non-mineralized granites of the Sierras de Córdoba (Coniglio et al., 2006).

Landscape corresponds to a highland environment strongly undulated with natural xeric vegetation with grasses, cacti and Bromeliaceae. Climatologically the study area is located within the semi-dry domain with water deficit in winter (Oggero and Arana, 2012). The anthropic intervention is low, mainly extensive livestock farming and extraction of fluorite opencast.

### 2.2 Field work

In November 2015, from each site, surface water was collected in 1 L plastic bottles (0.25 cm depth) to determine the concentration of major ions. Water temperature, pH, electrical conductivity, total dissolved solids, and salinity were measured *in situ*, using a digital multiparameter 35-Series 35425-10 tests (Oakton Instruments 625E Bunker Court Vernon Hills, IL 60061, USA). Dissolved oxygen was measured using a meter HD3030 (±1.5% FS). Furthermore, from each stream samples of periphyton from macrophytes, sediment and rocks were collected in order to analyze qualitatively algal communities in a multihabitat sample.

Tadpoles of *Boana cordobae* were collected using hand net from both streams. Tadpoles present nektonic habits and are usually found associated to the submerged vegetation (Altig and Johnston, 1989; McDiarmid and Altig, 1999). The care, treatment and sampling of animals used in this study followed the Animal Care Regulations of University National of Río Cuarto and state law “Protection and Conservation of Wild Fauna” (Argentina National Law Nº 22.421).

### 2.3 Laboratory work

The chemical analyses of surface water were performed in the area of Hydrology, National University of Río Cuarto, using standard methods (APHA-AWWA, 1999). The tadpoles were anesthetized by immersion in a solution at 0.05 % of MS222 and fixed in Phosphate Buffer. Development stage of each tadpole was recorded (Gosner, 1960) and only those between stages 30–35 were used to analyze the diet, since these Gosner stages are considered more stable in their morphological characters (Altig and McDiarmid, 1999; Zaracho et al., 2003). Furthermore, the total length (TL; length from the snout to the end of the tail), was measured using a manual SometInox Extra Vernier caliper (0.01 mm); and body weight, using an analytic balance OHAUS GT 200-S (0.00001 g).

Under a stereoscopic microscope, guts of five tadpoles of the HF-CN stream and seven of the LF-LV stream selected randomly were completely removed and the content of the first third were analyzed (Echeverria et al., 2007; Bionda et al., 2013 Pollo et al., 2015). The intestine content was analyzed quantitatively at 400× magnification, with algal organisms grouped taxonomically by genus. Specific bibliography for each particular group was used (Patrick and Reimer, 1966, 1975; Prescott, 1982; Komárek and Anagnostidis, 1998, 2005; Metzeltin et al., 2005; Komárek, 2013). For each sample, we counted three subsamples with slide and coverslip of 24 × 50 mm, following transects along the coverslip to determine cell densities (cells cm^−2^, based on Villafañe and Reid, 1995). The counting unit was the individual for unicellular, colonial and coenobial organisms and a 30 μm length for filaments (Cibils-Martina et al., 2017).

### 2.4 Data analysis

Water parameters were compared between sites using *t*-test. We computed Pearson's correlation coefficient to assess the relationship between body weight, total length and Gosner stage in tadpoles. Since the stage does not correlate with the variables total length and weight, we performed a *t*-test, to compare these variables between sites. Body weight of tadpoles was regressed on total length and the residuals were taken as an index of body condition (BC) of individuals (Wood and Richardson, 2009; Babini et al., 2016).

Rank-abundance curves were constructed to analyze and show graphically the dominance of certain algal groups and the presence of rare taxa, and whether these relationships changed between sites. We verified the collinearity of the parameters using Pearson's correlation coefficient and those highly correlated were eliminated from further analysis. Non-metric multidimensional scaling (NMDS), using the Bray-Curtis similarity coefficient (Quinn and Keough, 2002), was performed to visually describe differences in diet composition of tadpoles from both streams and the relation to environmental variables. For these analyses, we used square root transformed abundance to decrease the influence of abundant species. Permutational multivariate analysis of variance (PERMANOVA, Anderson, 2001; McArdle and Anderson, 2001) was performed to statistically test differences between groups, with 999 permutations. To check that differences between groups in terms of their centroids are not induced by differences in variances, we used analysis of multivariate homogeneity of group dispersions (PERMDISP, Anderson, 2001). The environmental variables with p ≤ 0.05 were graphed in NMDS.

Structural attributes of diets were calculated: density, richness, and Shannon diversity (H´) and evenness (J´) indices (calculated from food items densities and using log_10_ in the formula). Additionally, we assigned the algal genera to categories of functional traits according to Cibils et al. (2015): size classes, morphological guild, attachment mechanism, life-form and resources requirements (Supplementary material 1). We compared structural variables and the proportion of algae corresponding to different categories of functional traits using ANOVA. Validation of assumptions of ANOVA was performed reviewing standardized residuals vs. predicted, and the normal Q–Q plot of standardized residuals. Analyses for structural variables were performed using InfoStat (Di Rienzo et al., 2012). Multivariate analyses (NMDS and PERMANOVA) were performed in R version 3.3.2, using vegan library (Oksanen et al., 2013; R Core Team, 2013).

Rarefaction method was performed using EstimateS version 9.1.0 (Colwell, 2006) to standardize the samples and compare the average diet diversity and richness in tadpoles of both streams with different sample size.

## 3 Results

### 3.1 Environmental parameters and analysis of periphyton from macrophytes, sediment and rocks

Static analysis of environmental variables and the concentration of major ions are presented in Table 1. Water temperature (*t*-test: *t* = 0.35, p = 0.73) and dissolved oxygen (*t*-test: *t* = -2.07, p = 0.08) showed little variation between streams; whereas pH (*t*-test: *t* = -6.5, p˂0.001) showed higher values in LF-LV around alkaline range. Salinity (*t*-test: *t* = - 9.11, p˂0.01), conductivity (*t*-test: *t* = - 9.04, p˂0.001), total dissolved solids (*t*-test: *t* = - 9.83, p˂0.001) and most ion concentrations were higher in LF-LV. Fluoride was six times higher in HF-CN stream (*t*-test: *t* = 5.10, p˂0.05).

**Table 1:** Mean ± SE of the chemical, physical and ion concentration for each sampling site are given. Significant results of ANOVA are signaled with asterisks. Vectors of Non-metric multidimensional scaling analysis (NMDS) are provided.

	Sites		Vectors	
	LF-LV	HF-CN	NMDS1	NMDS2
Water Temperature (T°W)	18.24 ± 3.14	18.91 ± 3.46	0.22482	-0.9744
pH	8.38 ± 0.28	7.81 ± 0.30 **	-0.9918	0.1274 **
TDS (ppm)	85.03 ± 17.8	35.66 ± 14.35**	-0.9692	0.2462 **
Salinity (S) ppm	55.86 ± 10.65	26.48 ± 7.33 *	-0.9923	0.1240 **
Conductivity (C) μS/cm	117.8 ± 22.66	49.87 ± 20.66 **	-0.9493	0.3145 **
Dissolved Oxygen (O_2_) %	90.88 ± 10.25	91.02 ± 11.48	-0.9755	-0.2201*
CO_3_ mg/l	0.90 ± 1.80	0.00 ± 0.00	-0.6743	-0.7385
HCO_3_ mg/l	77.83 ± 9.48	38.75 ± 29.55	-0.8925	0.4510 *
Sulphates (SO4^=^) mg/l	23.25 ± 4.70	17.3 ± 12.24	-1.0000	-0.0028
Chloride (Cl^−^) mg/l	3.25 ± 0.70	7.9 ± 10.00	0.9457	0.3249
Sodium (Na^+^) mg/l	7.93 ± 0.96	9.75 ± 8.34	0.5219	0.853
Potassium (K^+^) mg/l	0.75 ± 0.26	0.53 ± 0.45	-0.9582	-0.2861
Calcium (Ca^++^) mg/l	15.8 ± 2.95	8.20 ± 6.35	-0.9471	0.3209 *
Magnesium (Mg^++^) mg/l	5.0 ± 1.9	2.45 ± 1.53	-0.7366	0.6763 *
Fluoride (F^−^) mg/l	0.33 ± 0.13	2.03 ± 0.66 *	0.9996	-0.0296 **

LF-LV: Los Vallecitos stream; HF-CN: Los Cerros Negros stream **p ≤ 0.001; *p ≤ 0.05

The qualitative analysis of periphyton from different substrates (Supplementary material 2) showed that LF-LV stream present higher richness (32 genera), while the HF-CN stream showed 22 genera. The HF-CN stream showed less richness of cyanobacteria and diatoms. In both streams, *Zygnema* sp. (Charophyta) and *Cymbella* sp. were the most abundant, and *Ulnaria* sp. (Bacillariophyta) predominates in HF-CN, forming rosette colonies.

### 3.2 Morphological measurements

**Fig. 1:**
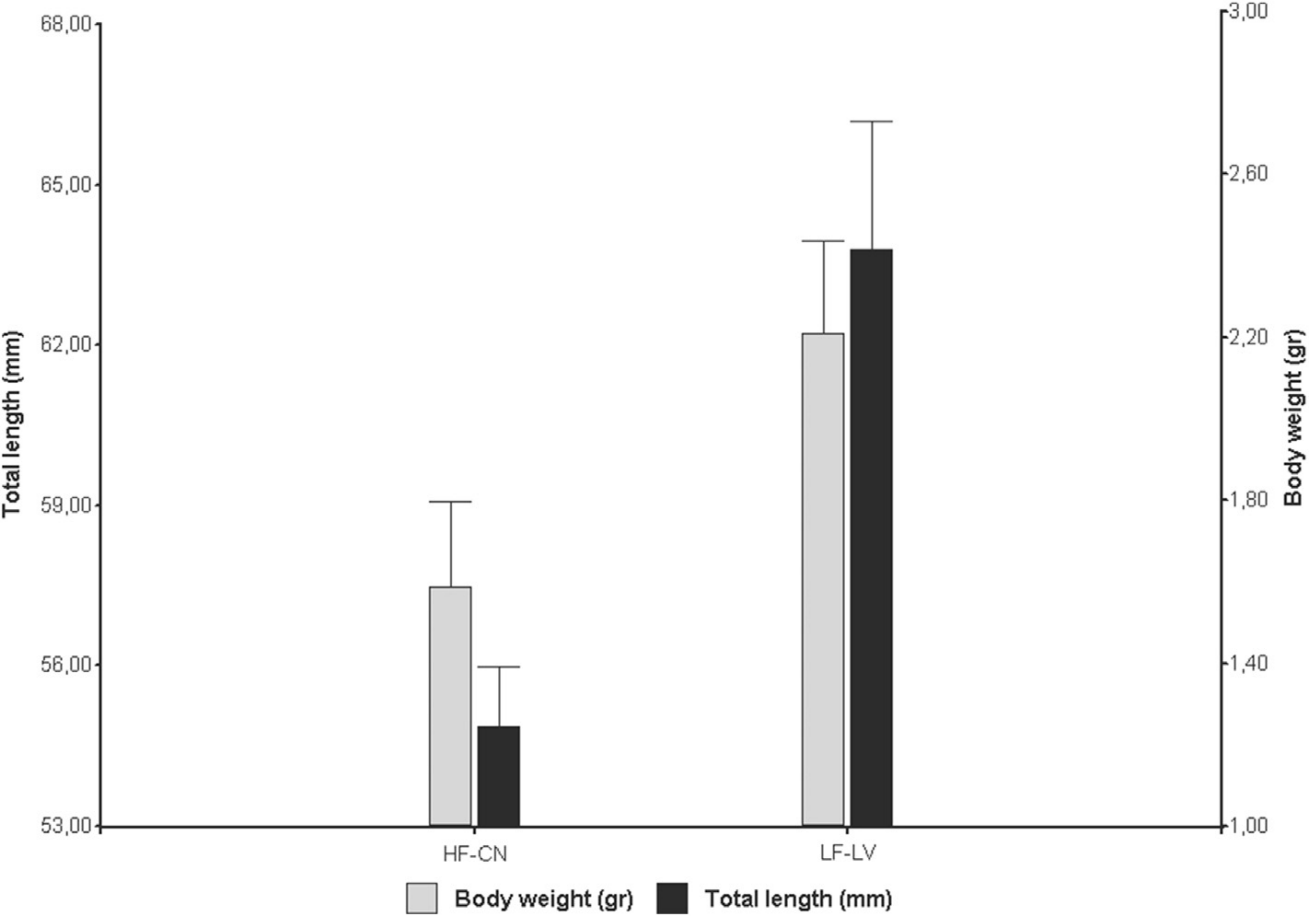
Total length and body weight of tadpoles of *Boana cordobae* per site. Values are mean ± SE.

The total length and body weight were not correlated with Gosner stage (p = 0.13; p = 0.72, respectively). For this reason, we compare the variables total length, body weight and body condition between sites using *t*-test*.* There was a significant difference in total length between sites (*t*-test: *t* = -2.96, p˂ 0.05), average total length was less in the HF-CN tadpoles (54.87 ± 2.48 mm) than in the LF-LV tadpoles (63.80 ± 6.34 mm). But, no significant differences in body weight (*t*-test: *t* = -1.94, p = 0.081) (Fig. 1) and body condition (*t*-test: *t* = -0.51, p = 0.62) were observed between sites.

### 3.3 Diet composition

A total of 90 taxa were identified in the diet of tadpoles from both streams, which mainly corresponded to microalgae, 48% Bacillariophyta, 14% Chlorophyta, 14% Cyanobacteria, 13% Charophyta, and 3% Euglenozoa. We also found some ciliates, testate amoebae and microfauna (nematodes, copepods, ostracods and rotifers), reaching 4% of total food items. In diets of tadpoles from HF-CN stream, Euglenozoa, ciliates, and microfauna were not registered, and the proportion of cyanobacteria was much lower than in tadpoles from LF-LV (Supplementary material 1). Also, in diets of tadpoles from HF-CN proportionally more filamentous chlorophytes and charophytes were registered, such as *Oedogonium* sp., *Spirogyra* sp., and *Mougeotia* sp.

**Fig. 2:**
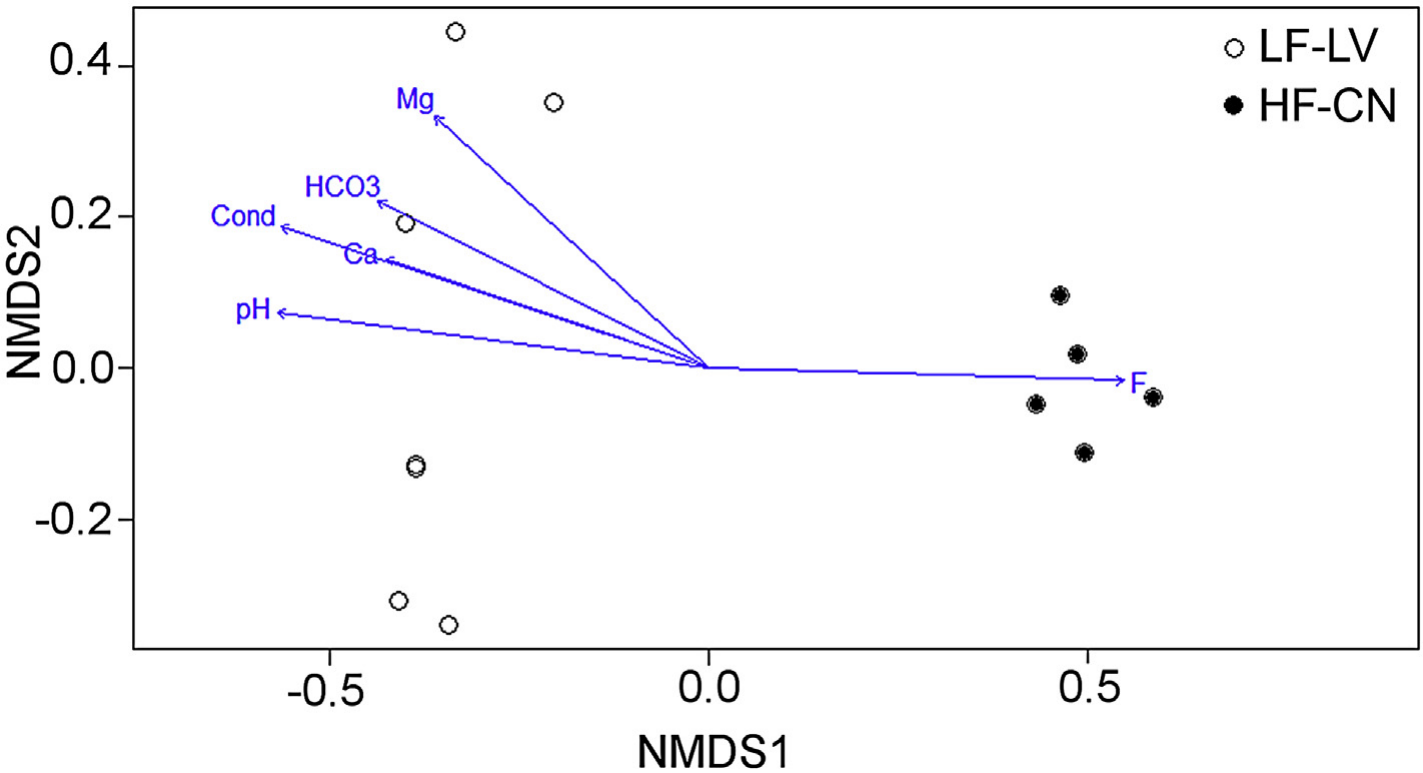
NMDS of the diet of tadpoles of *Boana cordobae* from high and low fluoride streams and environmental variables with significant association to each vector.

NMDS showed a clear differentiation in composition and structure of tadpoles diets from different streams (Fig. 2, stress = 0.03, PERMANOVA, F_1,10_ = 8.58, P = 0.001). This separations were not due to differences in dispersion within groups (PERMDISP, F_1,10_ = 2.61, P = 0.14). Furthermore, the biplot showed that fluoride influenced the separation of diets along with other variables (Table 1, Fig. 2).

**Fig. 3:**
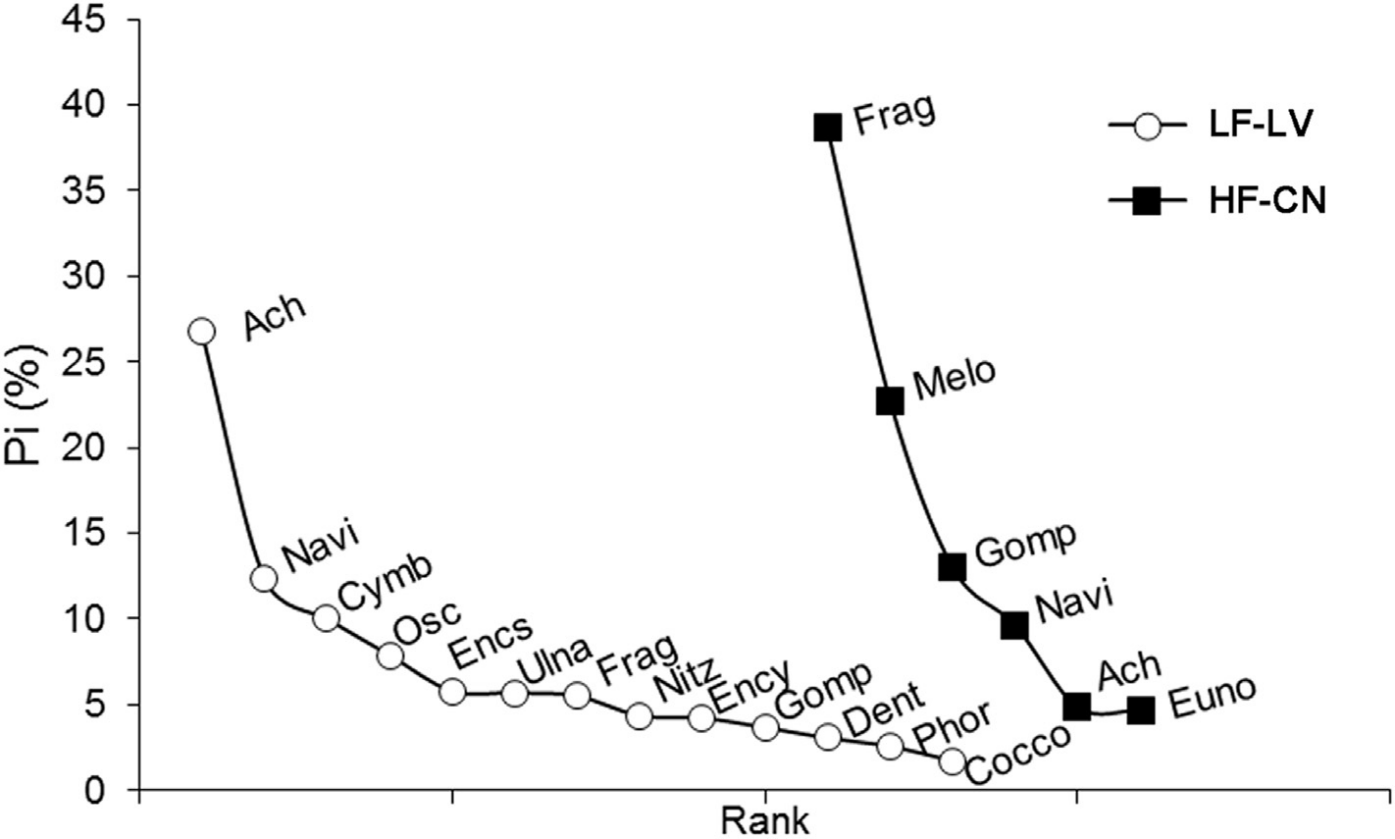
Rank-abundance curves of abundant taxa (P_i_ > 1%) in the diet of tadpoles of *Boana cordobae* from high and low fluoride streams. References: Ach: *Achnanthidium* sp., Navi: *Navicula* sp., Cymb: *Cymbella* sp., Osc: *Oscillatoria* sp., Encs: *Encyonopsis* sp., Ulna: *Ulnaria* sp., Frag: *Fragilaria* sp., Nitz: *Nitzschia* sp., Ency: *Encyonema* sp., Gomp: *Gomphonema* sp., Dent: *Denticula* sp., Phor: *Phormidium* sp., Cocco: *Cocconeis* sp., Melo: *Melosira* sp., Euno: *Eunotia* sp.

*Fragilaria* sp., *Gomphonema* sp. and *Melosira* sp. (Bacillariophyta) were the most abundant taxa in the diet of tadpoles of the HF-CN stream (Fig. 3). Instead, in the diet of tadpoles of the LF-LV stream, *Achnanthidium* sp., *Navicula* sp. and *Cymbella* sp. (Bacillariophyta) represented more than 10% of the diet. The most abundant species of these genera were *F. crotonensis* Kitton, *F. capucina* Desmazières, *G. pumilum* (Grunow) Reichardt & Lange-Bertalot, *G. parvulum* (Kützing) Kützing, *G. truncatum* Ehrenberg, *M. varians* C. Agardh, and long chains of *Eunotia major* (W. Smith) Rabenhorst were observed. In LF-LV the most abundant species were *A. minutissimum* (Kützing) Czarnecki, *N. radiosa* Kützing, *C. tumida* (Brébisson) VanHeurck, *C. excisa* var*. angusta* Krammer.

### 3.4 Structural attributes: density, richness, diversity and evenness

**Fig. 4:**
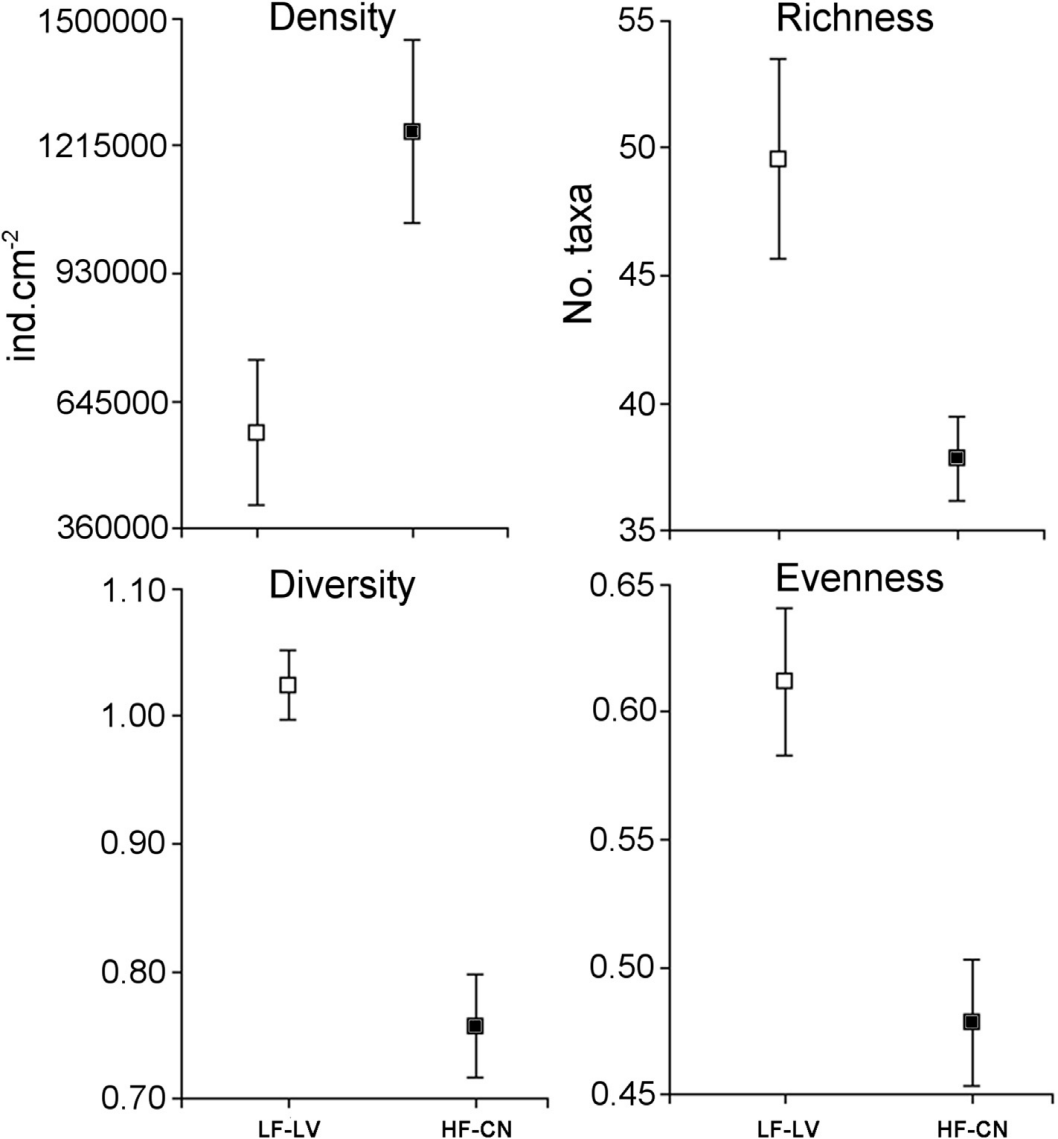
Structural attributes of the algal community in the diet of tadpoles of *Boana cordobae* from high (HF-CN: Los Cerros Negros stream, black) and low (LF-LV: Los Vallecitos stream, white) fluoride streams. Means and standard errors are shown.

Structural attributes of the algal community consumed by the tadpoles revealed that algal density was different between streams (ANOVA, F_1, 10_ = 6.91; P = 0.03, Fig. 4). Diet of tadpoles inhabiting HF-CN stream showed 2-fold higher density of algae. Richness, diversity and evenness of the diet showed differences in what was consumed according to each environment. Tadpoles from HF-CN stream showed diets with lower richness, diversity and evenness than individuals from LF-LV stream (ANOVA, richness: F_1, 10_ = 5.77, P = 0.04; diversity: F_1, 10_ = 32.70, P < 0.001; evenness: F_1, 10_ = 10.84, P = 0.008). Rarefaction method also showed that the diversity and richness of algae in the gut have significant differences between streams (diversity, *F*_1,10_ = 689.22, *P* < 0.0001; richness *F*_1,10_ = 23.63, P < 0.05).

### 3.5 Functional traits: size classes, morphological guilds, attachment mechanisms, life form and nutrient requirement

**Fig. 5:**
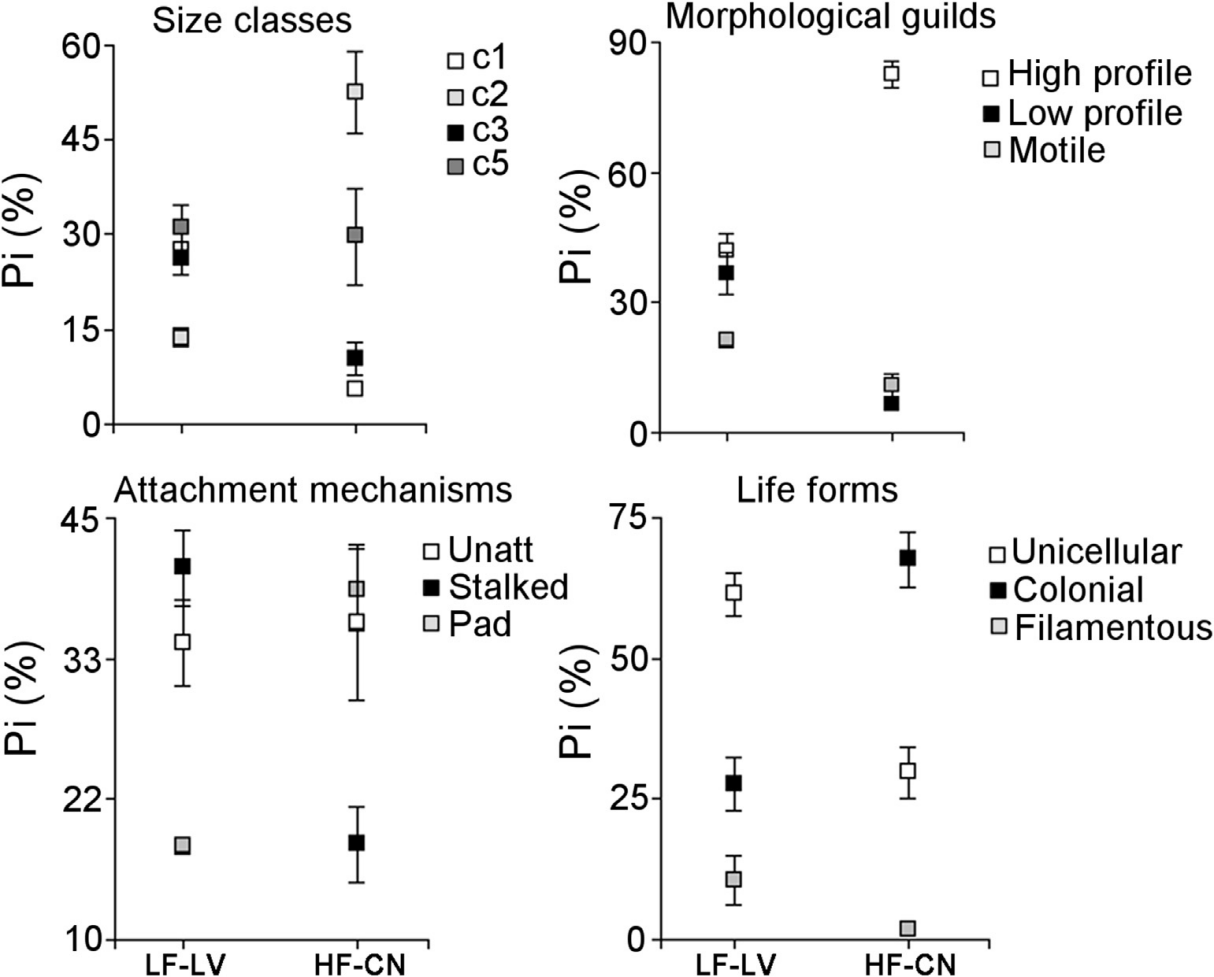
Relative abundance (P_i_) of taxa corresponding to selected functional traits in the diet of tadpoles of *Boana cordobae* from high and low fluoride streams. Mean and standard error are shown. For size classes, four of the five categories are shown (c1 comprises the smaller organisms and c5 the larger ones), for attachment mechanisms, three of the five and for life-forms, three of the four categories.

In relation to functional traits, a higher proportion of algae corresponding to c2 class size was observed in HF-CN diets (Table 2, Fig. 5), represented mainly by *Fragilaria* sp. and *Gomphonema* sp. Classes c1 and c3 showed lower values in HF-CN diets compared to LF-LV. Algae from c4 size class showed a low proportion (<1%) in both diets. Large algae (c5) were abundant in both streams diets, representing a 30% of the diet. Large algae were represented mainly by *Oscillatoria* sp. (42%), *Cymbella* sp. (19%), and *Ulnaria* sp. (13%), in diets from LF-LV stream, and by *Melosira* sp. (75%) and *Eunotia* sp. (16%) in diets from HF-CN stream. Regarding morphological guilds, high profile algae were predominant in HF-CN, while low profile algae and motile were more abundant in LF-LV. Regarding attachment mechanisms, algae with pads showed a higher proportion in HF-CN diets and stalked algae were more abundant in LF-LV. With respect to life forms, colonial algae were more abundant in HF-CN. Regarding nutrients requirements the proportion of sensitive and tolerant taxa were similar between treatments, around 50% of each category.

**Table 2:** One-way ANOVA results for proportions of functional traits of algae found in tadpole's diet from streams flowing through granitic rock with higher fluoride concentration (HF-CN) and metamorphic rock with lower fluoride concentration (LF-LV). For each variable, *F* value, degrees of freedom (*df*) of factor and error, *P* value are shown. Significant results are indicated in bold.

Trait	Variable	*df*	*F*	*P*
Size classes	c1	1, 10	21.93	**0.0009**
	c2	1, 10	46.69	**<0.0001**
	c3	1, 10	37.99	**0.0001**
	c4	1, 10	3.27	0.10
	c5	1, 10	0.03	0.86
Morphological Guilds	High profile	1, 10	57.56	**<0.0001**
	Low profile	1, 10	28.04	**0.0003**
	Motile	1, 10	12.09	**0.006**
Attachment mechanisms	Adnate	1, 10	0.59	0.46
	Pad	1, 10	51.88	**<0.0001**
	Stalked	1, 10	24.90	**0.0005**
	Holdfast	1, 10	22.05	**0.0008**
	Unattached	1, 10	0.06	0.81
Life forms	Unicellular	1, 10	27.81	**0.0004**
	Colonial	1, 10	32.19	**0.0002**
	Coenobial	1, 10	2.82	0.12
	Filamentous	1, 10	2.85	0.12
Nutrient requirement	Sensitive	1, 10	0.003	0.95
	Tolerant	1, 10	0.003	0.95

## 4 Discussion

### 4.1 Landscape composition: effects on the diet of tadpole

The concentration of ions in water is usually expressed as conductivity, an important factor in the distribution of biota (Cañedo Arguelles, 2013). In this sense, streams water can be classified as freshwater, with salt concentrations lower than 0.5 g/L, according to the Venice System (1959). Mining activity is one of the most common sources of salt addition to mountain rivers (Cañedo Arguelles, 2013). However, the low concentration of salts registered in this study indicates low anthropic activity in the basin. Thus, in absence of major anthropic influences, the main source of major ions in the stream is the weathering of the basin (Weinstein and Davison, 2004). Therefore, given that the level of fluoride in Los Cerros Negros (HF-CN) stream is above the limits (0.01–0.3 mg F/L) of unpolluted waters, the integrity of aquatic community could be affected (Camargo, 2003).

There is information available on fluoride toxicity in higher plants and algae, but laboratory data are contradictory and the environmental effects of this compound are not known (Camargo, 2003; Davison and Weinstein, 2006). To our knowledge, no published research have addressed *in situ* the response of algal community to high natural fluoride content in freshwater. Therefore, our research represents the first study to evaluate the influence of natural fluoride in lotic freshwater ecosystems on diet and morphology of tadpoles.

Algae are used to assess the quality of the environment (Sabater et al., 2007), especially diatoms due to their sensitivity to many environmental factors (Gómez and Licursi, 2001; Zampella et al., 2006; Seeligmann et al., 2008; Dunck et al., 2015). Fluoride has unfavorable effects for algae because it interacts negatively on photosynthesis, respiration, pigment synthesis, among other biological systems (Bhatnagar, 1997), causing chlorosis, necrosis, and morphological abnormalities in algae and aquatic plants (McPherson et al., 2014). Some experimental studies have shown that fluoride may suppress or intensify population growth of algae depending on its concentration, time of exposure, and algal species. For example, concentrations between 123 and 190 mg F/L showed 82% inhibition of growth of *Chlorella* sp. and *Selenastrum* sp. (Chlorophyta). However, other freshwater genera such as *Oscillatoria* sp. (Cyanobacteria), *Scenedesmus* sp. (Chlorophyta), *Cyclotella* sp. (Bacillariophyta) exposed to concentrations ˂50 mg F^−^/L is not affected (Camargo, 2003). Joy and Balakrishnan (1990) determined in laboratory that at concentrations of 10–100 mg F/L *Nitzschia palea* (Bacillariophyta) improved its growth, which could be due to a fluoride requirement for optimal growth. Nevertheless, Ali (2004) determined that with 4 mg F/L, it caused an inhibition in the growth of *N. palea* at low pH, given that F- become more toxic when crosses the membrane.

Our study of gut analysis showed that the two streams are different, according to the NMDS plot. The analysis of composition and structure of tadpole diets also allowed to differentiated the sites. Diversity, richness and evenness were lower in the diets of tadpoles collected from stream HF-CN. This suggested that a natural concentration of fluoride higher than 2 mg F/L could be deleterious for some algal species. A possible explanation could be that fluoride crosses the cell membrane, lodges in the cytoplasm and interacts with most of the cellular components, altering the general functioning of cell (Barbier et al., 2010).

### 4.2 Fluoride and morphological measures in tadpoles

Feeding of anuran tadpoles is generalist; therefore, their diets are indicative of the quality and abundance of the nutritional resource in their environment (Heyer, 1974; Lajmanovich, 2000; Rossa Feres et al., 2004; Bionda et al., 2011). However, in our study some more abundant algae were not consumed by the tadpoles. This could be due to the architecture of periphyton, making some algae easily consumed than others (Pollo et al., 2015). Food quality and ecological conditions may influence the age and size at metamorphosis of tadpoles (Carey and Bryant, 1995; Altig et al., 2007), thus affecting biological interactions such as competition and predation (Kupferberg, 1997).

In amphibians the body size can be strongly influenced by age and/or ambient conditions such as food supply, temperature, pollutants (Stepanyan et al., 2011; Cabrera-Guzmán et al., 2013; Babini et al., 2015). Our results showed that the total length of tadpoles of *B. cordobae* was not influenced by the larval stage. Therefore, the shortest total length recorded in larvae of the HF-CN stream could be due to high concentrations of fluoride, due to is a sensitive parameter a the effects of fluoride (Goh and Neff, 2003; Zhao et al., 2013; Chai et al., 2017; Pollo et al., 2016; Zhang et al., 2018). For example, Chai et al. (2017) found that total length of embryo of *Rana chensinensis* (Chordata, Ranidae) decreases with increasing levels of fluoride (≥0.7 mg F/L). The specific mechanism of inhibition may be the accumulation of fluoride in the body, which causes an imbalance in bone deposition and the remodeling activities that lead to skeletal fluorosis (Zhang et al., 2018). In addition, the metamorphosis of the tadpoles is delayed in the presence of high concentrations of fluoride (Chai et al., 2017). This effect is due to the fact that this element changes the histomorphology of the thyroid gland (Zhao et al., 2013) or alters thyroid hormone (Chen et al., 2016). In nature, the effect on total length of the tadpoles may result in an increased risk of predation and higher mortality. In addition, it can compromise persistence of the population because final size of the individuals also influences their reproduction and recruitment (Wilbur, 1980; Semlitsch et al., 1988; Gray and Smith, 2005).

However, when we analyzed biometric measurements together (total length and body weight) as an index of body condition, our results showed no differences in tadpoles between sites. Body condition is an indicator of the physiological state of the organism (Bagenal and Tesch, 1978; Jakob et al., 1996) directly related to diet, which is affected by the quality and quantity of food. At the same time, body weight could increase and decrease rapidly (Reading and Clarke, 1995). This could be explained by the nutritional quality of the algal community towards more or less inedible species. The diet of tadpoles of both streams showed higher abundance of chain-forming diatoms and large species, which can contribute to their body weight. In tadpoles from LF-LV stream this algae were represented by filamentous cyanobacteria and large diatoms, while in tadpoles from HF-CN large algae were long chains of diatoms *Melosira* sp. and *Eunotia* sp. In addition, there is a higher proportion of colonial and high profile algae in HF-CN site, which could have been more easily consumed (Cibils Martina et al., 2014). Another possible explanation could be that fluoride concentrations between 0.5 and 5 mg F/L increase gastrointestinal microbes, which contributes greatly to the health and digestive efficiency of tadpoles (Wang et al., 2019).

## 5 Conclusions

This study expands the limited number of research on the effect of natural fluoride on algae and amphibians. The ecotoxicological assessment of the effects of fluoride on the diet and morphology of tadpoles of *Boana cordobae*, added to effects on life history traits and body size in adult of this species reported for Otero et al. (2018), may be useful to predict the effects at the population level. Our study showed that the concentrations of major ions detected in streams are consistent with rock content (lithology of granitic and metamorphic rocks). We detect that landscape composition affects the structural attributes (density, richness, diversity and evenness) of tadpole diets, with some algal genera affected by higher concentrations of natural fluoride. However, the low algal richness registered in diet of tadpoles that inhabit the stream with high fluoride content did not affect the physiological state of them, since it was similar in both streams. The lower richness may have been compensated by the higher abundance of chain-forming diatoms in the diets of HF-CN tadpoles. In addition, it is important to consider the effect of fluoride on reduced body length of tadpoles, because it could have consequences at the population level. Nevertheless, more researches are needed that considers a greater number of samples and other amphibian species before broader generalizations are attempted.

## Declarations

### Author contribution statement

Favio Pollo, Luciana Cibils-Martina: Conceived and designed the experiments; Performed the experiments; Analyzed and interpreted the data; Contributed reagents, materials, analysis tools or data; Wrote the paper.

Manuel Otero, Mariana Baraquet, Pablo Grenat: Analyzed and interpreted the data; Contributed reagents, materials, analysis tools or data; Wrote the paper.

Nancy Salas, Adolfo Martino: Contributed reagents, materials, analysis tools or data.

### Funding statement

This work was supported by a grant from the Secretary of Research and Technology of National University of Río Cuarto (PPI 18/C475) and National Agency for Scientific and Technological Promotion FONCYT (BID-PICT 0932-2012; BID287 PICT 2533-2014). F.P., L.C., M.O., M.B., and P.G. thank CONICET - Argentina (Argentinean National Research Council for Science and Technology) for fellowships granted.

### Competing interest statement

The authors declare no conflict of interest.

### Additional information

No additional information is available for this paper.

## Appendix A Supplementary data

Online Resource 1 Online Resource 1

Online Resource 2 Online Resource 2

## References

[bib1] Ali G. (2004). Fluoride and aluminium tolerance in planktonic microalgae. Fluoride.

[bib2] Altig R., Johnston G.F. (1989). Guilds of anuran larvae: relationships among developmental modes, morphologies and habitats. Herpetol. Monogr..

[bib3] Altig R., McDiarmid R.W., McDiarmid, Alting (1999). Body plan. Development and morphology. Pp. 24-51. Tadpoles: the Biology of Anuran Larvae.

[bib82] Altig R., Whiles M.R., Taylor C.L. (2007). What do tadpoles really eat? Assessing the trophic status of an understudied and imperiled group of consumers in freshwater habitats. Freshwater Biol..

[bib4] Anderson M.J. (2001). A new method for non-parametric multivariate analysis of variance. Austral. Ecol..

[bib5] Antia N.J., Klut M.E. (1981). Fluoride addition effects on *Euryhaline phytoplankter* growth in nutrient-enriched seawater at an estuarine level of salinity. Bot. Mar..

[bib6] Antunes S.C., Castro B.B., Nunes B., Pereira R., Gonçalves F. (2008). *In situ* bioassay with *Eisenia andrei* to assess soil toxicity in an abandoned uranium mine. Ecotoxicol. Environ. Saf..

[bib7] APHA-AWWA-WEF, Lenore Clescerl, Greenberg Eaton (1999). Standard Methods for the Examination of Water and Wastewater.

[bib8] Arana M., Salas N., Correa A., di Tada I. (2003). Dieta de la larva de *Hyla pulchella cordobae* BARRIO, 1965 (Anura: Hylidae) en Pampa de Achala Córdoba Argentina. Bol. Asoc. Herpetol. Esp..

[bib9] Babini M.S., Bionda C.L., Salas N.E., Martino A.L. (2015). Health status of tadpoles and metamorphs of *Rhinella arenarum* (Anura, Bufonidae) that inhabit agroecosystems and its implications for land use. Ecotoxicol. Environ. Saf..

[bib10] Babini M.S., Cibils-Martina L., Luque E., Gari N., Salas N., Martino A.L. (2016). Anuran larvae diet from agroecosystem’s ponds: environmental quality and implications for their populations. J. Limnol..

[bib11] Bagenal T.B., Tesch F.W., Bagenal T.B. (1978). Age and growth. Methods for the Assessment of Fish Production in Fresh Waters.

[bib12] Ballarin L., Covre V., Masiero L., Casellato S. (2014). Immunotoxic effects of fluoride on the hemocytes of *Venerupis philippinarum*. Invertebr. Surviv. J..

[bib13] Barbier O., Arreola Mendoza L., Del Razo L.M. (2010). Molecular mechanisms of fluorite toxicity. Chem. Biol. Interact..

[bib14] Bhatnagar M. (1997). Fluoride Tolerance in Microalgae and its Ecological Implications.

[bib15] Bionda C., Gari N., Luque E., Salas N., Lajmanovich R., Martino A. (2012). Ecología trófica en larvas de *Rhinella arenarum* (Anura: Rhinellanidae) en agroecosistemas y sus posibles implicaciones para la conservación. Rev. Biol. Trop..

[bib16] Bionda C., Luque E., Gari N., Salas N.E., Lajmanovich R.C., Martino A.L. (2013). Diet of tadpoles of *Physalaemus biligonigerus* (Leiuperidae) from agricultural ponds in the central region of Argentina. Acta Herpetol..

[bib18] Cabrera-Guzmán E., Crossland M.R., Brown G.P., Shine R. (2013). Larger body size at metamorphosis enhances survival, growth and performance of young cane toads (*Rhinella marina*). PLoS One.

[bib19] Camargo J.A. (2003). Fluoride toxicity to aquatic organisms: a review. Chemosphere.

[bib20] Camargo J.A., Alonso Á. (2017). Ecotoxicological assessment of the impact of fluoride (F−) and turbidity on the freshwater snail *Physella acuta* in a polluted river receiving an industrial effluent. Environ. Sci. Pollut. Res..

[bib21] Cañedo Argüelles M., Kefford B.J., Piscart C., Prat N., Schäfer R.B., Schulz C.J. (2013). Salinisation of rivers: an urgent ecological issue. Environ. Pollut..

[bib22] Cardinale B.J., Matulich K.L., Hooper D.U., Byrnes J.E., Duffy E., Gamfeldt L. (2011). The functional role of producer diversity in ecosystems. Am. J. Bot..

[bib24] Chai L., Dong S., Zhao H., Deng H., Wang H. (2016). Effects of fluoride on development and growth of *Rana chensinensis* embryos and larvae. Ecotoxicol. Environ. Saf..

[bib25] Chai Y., Kim D., An Y.J. (2016). Effect of fluoride on the cell viability, cell organelle potential, and photosynthetic capacity of freshwater and soil algae. Environ. Pollut..

[bib26] Chai L., Wang H., Zhao H., Dong S. (2017). Chronic effects of fluoride exposure on growth, metamorphosis, and skeleton development in *Bufo gargarizans* larvae. Bull. Environ. Contam. Toxicol..

[bib27] Chen J.J., Xue W.J., Cao J.L., Song J., Jia R.H., Li M.Y. (2016). Fluoride caused thyroid endocrine disruption in male zebrafish (*Danio rerio*). Aquat. Toxicol..

[bib28] Cibils L., Principe R., Márquez J., Gari N., Albariño R. (2015). Functional diversity of algal communities from head water grass land streams: how does it change following afforestation?. Aquat. Ecol..

[bib29] Cibils-Martina L., Márquez J., Principe R., Gari N., Albariño R. (2014). Does grazing change algal communities from grassland and pine afforested streams?: a laboratory approach. Limnologica.

[bib30] Cibils-Martina L., Márquez J., Principe R., Gari N., Albariño R. (2017). Pine afforestation affects key primary producers in mountain grass land streams in Córdoba, Argentina. N. Z. J. Mar. Freshw. Res..

[bib31] Colwell R.K. (2006). EstimateS: statistical estimation of species richness and shared species from samples. http://purl.oclc.org/estimates.

[bib32] Coniglio J., D`Eramo F., Pinotti L., Demartis M., Petrelli H. (2006). Magmatismo devónico de las sierras de córdoba: fuente posible de flúor de las mineralizaciones mesozoicas el ejemplo del batolito cerro áspero.

[bib33] Crane M., Burton G.A., Culp J.M., Greenberg M.S., Munkittrick K.R., Ribeiro R., Salazar M.H., St-Jean S.D. (2007). Review of aquatic in situ approaches for stressor and effect diagnosis. Integr Environ Asses.

[bib34] Davison A.W., Weinstein L.H. (2006). Some problems relating to fluorides in the environment: effects on plants and animals. Adv. Fluorine Sci..

[bib35] Di Rienzo J.A., Casanoves F., Balzarini M.G., Gonzalez L., Tablada M., Robledo C.W. (2012). InfoStatversión 2012 GrupoInfoStat, FCA.

[bib36] Dunck B., Rodrigues L., Bicudo D.C. (2015). Functional diversity and functional traits of periphytic algae during a short-term successional process in a Neotropical floodplain lake. Braz. J. Biol..

[bib37] Echeverria D.D., Volpedo A.V., Mascitti V.I. (2007). Diet of tadpoles from a pond in Iguazu National Park, Argentina. Gayana.

[bib38] Franklin N.M., Stauber J.L., Markich S.J., Lim R.P. (2000). pH-dependent toxicity of copper and uranium to a tropical freshwater alga (Chlorella sp.). Aquat. Toxicol..

[bib39] Frost D.R. (2018). Amphibian Species of the World. http://research.amnh.org/herpetology/amphibia/.

[bib40] Gómez N., Licursi M. (2001). The pampean Diatom Index (IDP) for assessment of river and streams in Argentina. Aquat. Ecol..

[bib41] Gosner K.L. (1960). A simplified table for staging anuran embryos and larvae with notes on identification. Herpetologica.

[bib42] Hekman W.E., Budd K., Palmer G.R., MacArthur J.D. (1984). Responses of certain freshwater planktonic algae to fluoride. J. Phycol..

[bib43] Jakob E.M., Marshall S.D., Uetz G.W. (1996). Estimating fitness: a comparison of body condition indices. Oikos.

[bib44] Joy C.M., Balakrishnan K.P. (1990). Effect of fluoride on axenic cultures of diatoms. Water Air Soil Pollut..

[bib45] Komárek J., Büdel B., Gärtner G., Krienitz L., Schagerl M. (2013). Cyanoprokaryota 3. Teil: Heterocytous genera. Süβwasserflora von Mitteleuropa 19/3.

[bib46] Komárek J., Anagnostidis K., Ettl H., Gärtner G., Heynig H., Mollenhauer D. (1998). Cyanoprokaryota 1. Teil: chroococcales. Süβwasserflora von Mitteleuropa 19/1.

[bib47] Komárek J., Anagnostidis K., Büdel B., Krienitz L., Gärtner G., Schagerl M. (2005). Cyanoprokaryota 2. Teil: oscillatoriales. Süβwasserflora von Mitteleuropa 19/2.

[bib48] Kupferberg S.J. (1997). The role of larval diet in anuran metamorphosis. Am. Zool..

[bib50] McArdle B.H., Anderson M.J. (2001). Fitting multivariate models to community data: a commenton distance-based redundancy analysis. Ecology.

[bib51] McDiarmid R.W., Altig R. (1999). Tadpoles: the Biology of Anuran Larvae.

[bib52] McPherson C.A., Lee D.H., Chapman P.M. (2014). Development of a fluoride chronic effects benchmark for aquatic life in freshwater. Environ. Toxicol. Chem..

[bib53] Metzeltin D., Lange-Bertalot H., García-Rodríguez F. (2005). Diatoms of Uruguay. Compared with Other Taxa from South America and Elsewhere.

[bib55] Oggero A.J., Arana M.D. (2012). Inventario de las plantas vasculares del sur de la zona serrana de Córdoba, Argentina. HOEHNEA.

[bib56] Oksanen J., Guillaume Blanchet F., Kindt R., Legendre P., Minchin P.R., O’Hara R.B., Simpson G.L., Solymos P., Henry M., Stevens H., Wagner H. (2013). Vegan: community ecology package.

[bib57] Oliveira L., Antia N.J., Bisalputra T. (1978). Culture studies on the effects from fluoride pollution on the growth of marine phytoplankters. J. Fish. Res. Board Can..

[bib58] Otero M.A., Pollo F.E., Grenat P.R., Salas N.E., Martino A.L. (2018). Differential effects on life history traits and body size of two anuran species inhabiting an environment related to fluorite mine. Ecol. Indicat..

[bib59] Patrick R., Reimer C.W. (1966). The diatoms of the United States exclusive of Alaska and Hawai. Vol. 1. Acad. Nat. Sci. Phila. Monogr..

[bib60] Patrick R., Reimer C.W. (1975). The diatoms of the United States exclusive of Alaska and Hawai. Vol. 2. Acad. Nat. Sci. Phila. Monogr..

[bib61] Pollo F.E., Cibils L., Bionda C.L., Salas N.E., Martino A.L. (2015). Trophic ecology of syntopic anuran larvae, *Rhinella arenarum* (Anura: Bufonidae) and *Hypsiboas cordobae* (Anura: Hylidae): its relation to the structure of periphyton. Ann Limnol Int J Lim.

[bib62] Pollo F.E., Grenat P.R., Otero M.A., Salas N.E., Martino A.L. (2016). Assessment in situ of genotoxicity in tadpoles and adults of frog *Hypsiboas cordobae* (Barrio 1965) inhabiting aquatic ecosystems associated to fluorite mine. Ecotoxicol. Environ. Saf..

[bib64] Prescott G.W. (1982). With an Illustrated Key to the Genera of Desmids and Freshwater Diatoms. Algae of the Western Great Lakes Area.

[bib65] Quinn G.P., Keough M.J. (2002). Experimental Design and Data Analysis for Biologists.

[bib66] R CoreTeam (2013). R: a Language and Environment for Statistical Computing. http://www.R-project.org/.

[bib67] Sabater S., Guasch H., Ricart M., Romaní A., Vidal G., Klünde C., Schmitt-Jansen M. (2007). Monitoring the effect of chemicals on biological communities. The biofilm as an interface. Anal. Bioanal. Chem..

[bib68] Santos F.J., Protázio A.S., Moura C.W., Juncá F.A. (2016). Diet and food resource partition among benthic tadpoles of three anuran species in Atlantic Forest tropical streams. J. Freshw. Ecol..

[bib69] Seeligmann C., Maidana N.I., Morales M. (2008). Diatomeas (Bacillariophyceae) de humedales de altura de la Provincia de Jujuy-Argentina. Bol. Soc. Argent. Bot..

[bib71] Shi X., Wang R., Zhuang P., Zhang L., Feng G. (2013). Fluoride retention after dietary fluoride exposure in S iberian sturgeon A cipenser baerii. Aquacult. Res..

[bib72] Stepanyan I.E., Tsarukyan A.S., Petrov Y.P. (2011). Effect of molybdenum, chrome and cadmium ions on metamorphosis and erythrocytes morphology of the marsh frog *Pelophylax ridibundus* (Amphibia: Anura). J Environ Sci Technol.

[bib73] Venice system (1959). The final resolution of the symposium on the classification f brackish waters. Arch. Oceanogr. Limnol..

[bib74] Villafañe V.E., Reid F.M.H., Alveal K., Ferrario M.E., Oliveira E.C., Sar E. (1995). Métodos de microscopia para la cuantificación del fitoplancton. Manual de Métodos Ficológicos.

[bib75] Weinstein L.H., Davison A. (2004). Fluorides in the Environment: Effects on Plants and Animals.

[bib76] Wood S.L., Richardson J.S. (2009). Impact of sediment and nutrient inputs on growth and survival of tadpoles of the Western Toad. Freshw. Biol..

[bib77] Zampella R., Bunnell J.F., Laidig K.J., Procopio N.A. (2006). Using multiple indicators to evaluate the ecological integrity of a coastal plain stream system. Ecol. Indicat..

[bib78] Zaracho V.H., Céspedez J.A., Álvarez B.B. (2003). Descripción de caracteres morfológicos en larvas prometamórficas de *Physalaemus biligonigerus* (Anura: Leptodactylidae). Facena.

[bib79] Zhang Y., Xie L., Li X., Chai L., Chen M., Kong X., Wang Q., Liu J., Zhi L., Yang C., Wang H. (2018). Effects of fluoride on morphology, growth, development, and thyroid hormone of Chinese toad (*Bufo gargarizans*) embryos. Environ. Mol. Mutagen..

[bib80] Zhao H., Chai L., Wang H. (2013). Effects of fluoride on metamorphosis, thyroid and skeletal development in *Bufo gargarizans* tadpoles. Ecotoxicology.

[bib81] Zuo H., Chen L., Kong M., Qiu L., Peng L., Wu P. (2018). Toxic effects of fluoride on organisms. Life Sci..

