# Supplementary material for: Anuran tadpoles inhabiting a fluoride-rich stream: diets and morphological indicators
Source: Heliyon. 2019 Jun 26;5(6):e02003. doi: 10.1016/j.heliyon.2019.e02003 (PMC6600003; doi:10.1016/j.heliyon.2019.e02003)
Supplement: Online Resource 2 [file mmc2.docx]

Taxonomic list of periphyton from macrophytes, sediment and rocks

Title article: Anuran tadpoles inhabiting fluoride-rich streams: diet/feeding and morphological indicators

Journal: Heliyon

Authors: Favio E. Pollo ^1,3^; Luciana Cibils-Martina ^2,3^; Manuel A. Otero ^1,3^; Mariana Baraquet ^1,3*^; Pablo R. Grenat ^1,3^; Nancy E. Salas^1^, Adolfo L. Martino^1^

^1-^ Ecología, Departamento de Ciencias Naturales, Facultad de Ciencias Exactas, Físico-Químicas y Naturales, UNRC, ruta 36km 601, Río Cuarto, Córdoba, Argentina. Tel: 011-0358-4673167. Fax: 011-0358-4676230.

^2-^ Botánica Sistemática, Departamento de Ciencias Naturales, Facultad de Ciencias Exactas, Físico-Químicas y Naturales, UNRC.

^3^ - Consejo Nacional de Investigaciones Científicas y Técnicas (CONICET), Argentina

*Corresponding author e-mails: mbarquet@exa.unrc.edu.ar

| Qualitative analysis of samples of macrophytes, sediment and rocks for the sites sampled | | |
| --- | --- | --- |
| **Taxa** | **Low fluoride- Los Vallecitos stream** | **High fluoride – Los Cerros Negro stream** |
| **Cyanobacteria** |  |  |
| *Aphanocapsa* sp | X | - |
| *Coleodesmium* sp | X | - |
| *Merismopedia* sp | X | - |
| *Oscillatoria* sp | X | X |
| **Chlorophyta** |  |  |
| *Ankistrodesmus* sp | X | - |
| *Bulbochaete* sp | - | X |
| *Desmodesmus* sp | X | - |
| *Monoraphidium* sp | X | X |
| *Oedogonium* sp | X | X |
| *Oocystis* sp | X | - |
| *Scenedesmus* sp | X | X |
| *Ulothrix* sp | X | X |
| **Charophyta** |  |  |
| *Closterium* sp | - | X |
| *Cosmarium* sp | X | X |
| *Desmidium* sp | - | X |
| *Euastrum* sp | - | X |
| *Mougeotia* sp | X | X |
| *Pediastrum* sp | X | - |
| *Spirogyra* sp | X | X |
| *Staurastrum* sp | X | X |
| *Zygnema* sp | X | X |
| **Bacillariophyta** |  |  |
| *Achnanthidium* sp | X | X |
| *Amphipleura* sp | X | - |
| *Amphora* sp | X | - |
| *Cocconeis* sp | X | - |
| *Cymbella* sp | X | X |
| *Diploneis* sp | X | - |
| *Encyonema* sp | X | - |
| *Encyonopsis* sp | X | - |
| *Eunotia* sp | - | X |
| *Fragilaria* sp | X | X |
| *Gomphonema* sp | X | X |
| *Karayevia* sp | X | - |
| *Melosira sp* | X | - |
| *Navicula* sp | X | X |
| *Nitzschia sp* | X | X |
| *Ulnaria* sp | X | X |
| *Total* | 32 | 22 |
